# Aesthetic chills cause an emotional drift in valence and arousal

**DOI:** 10.3389/fnins.2022.1013117

**Published:** 2023-03-07

**Authors:** Abhinandan Jain, Felix Schoeller, Adam Horowitz, Xiaoxiao Hu, Grace Yan, Roy Salomon, Pattie Maes

**Affiliations:** ^1^MIT Media Lab, Cambridge, MA, United States; ^2^The Gonda Multidisciplinary Brain Research Centre, Bar-Ilan University, Ramat Gan, Israel; ^3^Institute for Advanced Consciousness Studies, Santa Monica, CA, United States

**Keywords:** chills, emotion, valence, arousal, emotional drift, ChillsDB, interindividual differences, synchronization

## Abstract

Aesthetic chills are an embodied peak emotional experience induced by stimuli such as music, films, and speeches and characterized by dopaminergic release. The emotional consequences of chills in terms of valence and arousal are still debated and the existing empirical data is conflicting. In this study, we tested the effects of ChillsDB, an open-source repository of chills-inducing stimuli, on the emotional ratings of 600+ participants. We found that participants experiencing chills reported significantly more positive valence and greater arousal during the experience, compared to participants who did not experience chills. This suggests that the embodied experience of chills may influence one’s perception and affective evaluation of the context, in favor of theoretical models emphasizing the role of interoceptive signals such as chills in the process of perception and decision-making. We also found an interesting pattern in the valence ratings of participants, which tended to harmonize toward a similar mean after the experiment, though initially disparately distributed. We discuss the significance of these results for the diagnosis and treatment of dopaminergic disorders such as Parkinson’s, schizophrenia, and depression.

## 1. Introduction

Aesthetic chills (thereafter “chills”) are a peak emotional response characterized by a feeling of cold down the spine, sometimes accompanied by goosebumps (e.g., on the arms). While chills are a growing topic of study, evidence is still conflicting in regards to the emotional consequences of chills and interindividual differences (see e.g., Panksepp, 1995; Grewe et al., 2011; Zickfeld et al., 2020; de Fleurian and Pearce, 2021). Specifically, the causes and consequences of chills in terms of valence (i.e., hedonic tone or affective quality) and arousal (i.e., the level of autonomic activation) are still unclear (de Fleurian and Pearce, 2021). Chills have been linked to a wide range of emotions, both positive and negative, as well as general arousal response (Maruskin et al., 2012). Brain studies suggest chills engage reward-related brain regions, in the striatum and prefrontal cortex (Blood and Zatorre, 2001) specifically rewarding dopamine release in the caudate nucleus and nucleus accumbens (Salimpoor et al., 2011). While chills tend to produce physiological arousal and reward, the underlying mechanism, consequences and downstream effects are still unclear. In order to address these questions, we tested the emotional reaction of 600+ participants to ChillsDB, an open-source database of chills stimuli, searching for interindividual differences and variations in the ratings of valence and arousal based on whether or not the participants experienced chills.

Chills seem to be a universal emotional phenomenon found across human cultures and languages (McCrae, 2007). As a marker of human peak experiences across the arts, sciences, and world religions (Schoeller, 2015a), chills can be generated by a wide range of media: music, films, paintings, poetry, science, mathematics, religion, and rituals (Schoeller, 2015b). Among others, they have psychological consequences on pleasure and reward (Blood and Zatorre, 2001; Schoeller and Perlovsky, 2016), prosocial tendencies and altruism (Fukui and Toyoshima, 2014), meaning-making (Schoeller, 2015a), attention, memory, and cognitive function (Sarasso et al., 2020). Their relationship to physiologic factors includes heart rate (Sumpf et al., 2015), pupil dilatation (Laeng et al., 2016), skin conductance (Grewe et al., 2009), and indeed muscle contractions (McPhetres and Zickfeld, 2022). Research has investigated chills’ relationship to emotional valence and arousal. For example, the emotional features of chill-eliciting music have been examined (Panksepp, 1995; Grewe et al., 2011; de Fleurian and Pearce, 2021). When analyzing approximately 1,000 musical stimuli, de Fleurian and Pearce found that chills music was on average more negative in valence, in accordance with previous findings that chills are more frequently associated with perceived sadness (Panksepp, 1995). Salimpoor et al. (2011) and Laeng et al. (2016) found that physiological markers of arousal predicted chills when listening to music. The study of the adjacent emotion of “being moved” by Zickfeld et al. (2020) found that experiences rated as “very moving” resulted in less arousal compared to emotional experiences rated as “sad.” The evidence further suggests that there may be interindividual differences in chills induced by personality and gender. It seems that females experience musical chills more often than males (Panksepp, 1995; Kunkel et al., 2008). Here again, existing evidence is conflicting as Goldstein’s (1980), Rickard’s (2004), and Grewe et al.’s (2009) found no significant difference in terms of gender. To our knowledge, no age differences have been reported thus far (Goldstein, 1980; Panksepp, 1995; Grewe et al., 2009). In terms of personality, chills have been found to be a good predictor of the personality trait Openness to Experience (McCrae, 2007), specifically item 188 of NEO Personality Inventory (NEO PI), which measures chills in response to music and the arts (McCrae, 2007). This accumulated evidence calls for large scale studies of the chills phenomenon, mapping stimuli, participants’ states and traits, and context. The present study is a first attempt to better characterize the emotion of chills by examining a large corpus of chills stimuli (ChillsDB) constituted by parsing social media (YouTube and Reddit). Based on the prior research, we expect that (1) chills should have an effect on valence and arousal, (2) there should be interindividual differences in chills demographics (genre, ethnicity, and age), and (3) the Revised NEO Personality Inventory (NEO-PI-R) and Item 188, in particular, should predict for chills (McCrae, 2007; Krishnakumar and Schoeller, 2019).

## 2. Materials and methods

### 2.1. Stimuli

We used the top 50 videos of ChillsDB, an open-source database of validated audiovisual stimuli eliciting aesthetic chills (goosebumps, psychogenic shivers) in a US population (Schoeller et al., 2022b). The database consists of 204 chills-eliciting videos in three categories: music, film, and speech (see Table 1). ChillsDB was built using an ecologically-valid method for harnessing chills stimuli “in the wild” by searching for mentions of somatic markers in user comments using algorithms to parse social media platforms (YouTube and Reddit).

**TABLE 1:** A gold standard for aesthetic chills: the top 10 validated videos from our study, including the top three for each category (film, music, and speech).

Video title	Type	Video description	Average chills reported
Jurassic World	Movie	The final battle between Indominus Rex, Tyrannosaurus Rex, and Velociraptors as it appeared in the movie “Jurassic World” (2015).	3.90
The Hunger Games	Movie	Fan made video of “The Hanging Tree” from the score of “The Hunger Games.” The music was composed by James Newton Howard.	4.18
Naruto	Movie	An important battle in the widely popular Japanese manga series written and illustrated by Masashi Kishimoto.	2.90
Miserere	Music	Allegri’s Miserere is written for two choirs, who alternate phrases and then unite for a final resolution.	2.60
Dream	Speech	A medley of motivational speeches by speakers such as Les Brown, Eric Thomas, and Will Smith.	3.00
Dropout Wisdom	Speech	A speech by Dr. Rick Rigsby about lessons learned from his father.	3.10
Interstellar	Movie	Fan made video with Interstellar’s Hans Zimmer theme. Chills occur at the line “Because my dad promised me.”	3.20
Unbroken	Speech	Fan made video with motivational speeches by Les Brown, Eric Brown, and Steve Jobs.	2.50
Disney Heroes	Movie	Fan made video of Disney heroes singing in their native languages, with medley images of their stories.	3.90
Giving	Movie	“Giving” is a 3 min Thai TV commercial by TrueMove mobile company. Concept by Panu Meepaibul.	3.06

All stimuli have a probability ≥ 0.8 to elicit chills in a US population (from Schoeller et al., 2022a).

### 2.2. Procedure

We conducted an online study on Prolific crowdsourcing platform^1^ to evaluate the emotional effect of chill-inducing videos. Participants were first screened for neurologic disorders and randomly assigned to one of ChillsDB top 50 videos. Before the video, they were given a definition of “the feeling of emotional chills and shivers” as “the feeling of cold down your spine that are NOT related to temperature or sickness but that are caused by some strong emotions.” Participants were asked to report their age, gender, ethnicity, frequency of daily chills (1 - never; 5 - always), NEO item 188 score (“Sometimes when I am reading poetry or looking at a work of art, I feel a chill or wave of excitement”) (1 - Strongly Disagree; 5- Strongly Agree). Following a circumplex model of emotion (Russell, 1980), participants were asked to report their current mood in feeling “Extremely Unpleasant” to “Extremely Pleasant” for the valence rating, “Extremely Calm” to “Extremely Excited” for the arousal rating, “Extremely Sleepy” to “Fully Awake” for sleepiness rating on a 10 point Likert scale before the video. Participants were then exposed to the audiovisual stimulus from the database and asked to report whenever they experienced chills by pressing a large button on the right side of the screen. Only once the stimulus is over are they allowed to continue to the next part of the study. After the exposure they were asked to report the intensity of the chills, and rate their experience in terms of valence and arousal all on a five point Likert scale. Finally, we conclude by asking the final five items of the NEO questionnaire (“Do you feel you do not have good imagination,” “Are you quick to understand things,” “Do you use difficult words,” “Do you spend time reflecting things,” and “Are you full of ideas.”) on a five point Likert scale.

To ensure the quality of data we implemented two attention checks, one before the stimulus and one after, to determine if participants were paying attention to questions (Oppenheimer et al., 2009). The attention checks constituted selecting the correct response to a described question, e.g., “Please select ‘strongly agree’ for this question.” Participants were compensated based on time spent in the study at an approximately hourly rate of USD 11.72. Each experiment lasted approximately 10 min and was well-received by the participant. Some of the participants even wrote to the authors a personal email to thank them for the experience (e.g., “I just wanted to take a moment and tell you that I thoroughly enjoyed this study and found it also to be a unique experiment”).

### 2.3. Participants

Participants were recruited on the Prolific platform and were screened for psychiatric conditions or neurologic disorders. 660 subjects participated in the experiment (Mean age = 33.6, 50% males, 49.5% female, and 0.5% = other). We removed 100 participants who reported an aberrant proportion of chills (*N* > 10) and did not fulfill the two attention checks. All the participants reside in the United States of America and practice the English language as their first language. 75.7 White, 7.9 Multiracial, 8.4 Asian, 3.6 African American, 3.9 Hispanic, and 0.5% Others.

### 2.4. Ethics

The experiment is in compliance with the Helsinki Declaration. The study was approved by the Committee on the Use of Humans as Experimental Subjects at MIT. All participants gave their voluntary informed consent and we followed the Ethics Code of the American Psychological Association. All participants were informed about the purpose of the research, their right to decline to participate and to withdraw from the experiment, and the limits of confidentiality. We also provided them with a contact for any questions concerning the research and with the opportunity to ask any questions regarding the phenomenon under study (aesthetic chills) and receive appropriate answers. All participants reacted positively to the experiment and were thankful for the opportunity to learn about the phenomenon.

### 2.5. Reviewer disclosure

Following the standard reviewer disclosure request endorsed by the Center for Open Science (Lilienfeld, 2017). We confirm to have reported all measures, conditions, data exclusions, and how we determined our sample sizes.

## 3. Results

### 3.1. Chills participants

**FIGURE 1:**
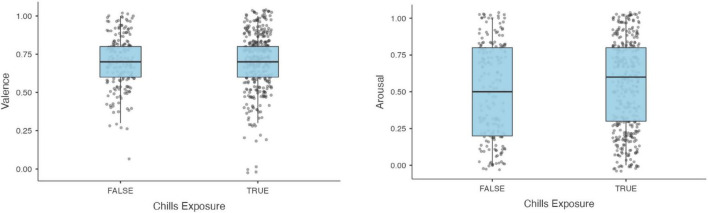
Arousal and valence ratings prior to exposure to the stimulus are normalized across participants, independently of whether they reported chills. Hence, prior valence and arousal do not seem to influence the probability of participants to experience chills.

We then examined interindividual responses in chills (Table 2). A total of 369 participants reported chills (66%). These participants who reported more chills (*M* = 35.7, SD = 13.5), compared to those who did not (*M* = 38.7, SD = 14.7) were significantly younger [*t*(558) = 2.41, *p* < 0.01]. Participants who reported chills experienced chills more frequently in everyday life (*M* = 1.3, SD = 0.7) as compared to those who did not report chills during the experiment (*M* = 1.1, SD = 0.7), this difference is statistically significant [*t*(558) = −2.83, p* = 0.005]. Furthermore, participants who reported chills (*M* = 2.1, SD = 1.1) had significantly higher scores on the NEO Item 188 [*t*(558) = −4.28, p* < 0.001] than participants who did not report chills (*M* = 1.6, SD = 1.3). Prior subjective ratings of valence and arousal did not seem to influence the probability of participants to experience chills (Figure 1).

**TABLE 2:** Independent samples *T*-test for age, daily occurrences of chills and NEO item 188.

		Statistic		df	*P*
Age	Student’s *t*	2.41		558	0.016
Daily chills	Student’s *t*	-2.83		558	0.005
NEO Item 188	Student’s *t*	-4.28	a	558	<0.001

^a^Levene’s test is significant (p < 0.05), suggesting a violation of the assumption of equal variances.

ANOVA was performed to test the difference in chills between ethnicities. ANOVA revealed that there was not a statistically significant difference in chills between the groups [*F*(5) = 1.23, *p* = 0.29]. We also performed an ANOVA to search for a difference in chills across gender, the test revealed that there was not a significant difference between genders [*F*(3) = 0.55, *p* = 0.64]. We did not find any statistical difference in terms of post cumulative NEO score between participants who experienced chills and those who did not [*t*(558) = −0.6, *p* > 0.55]. We found that prior exposure to the stimulus did not affect chills prevalence [*t*(558) = −1.15, *p* > 0.25].

### 3.2. Effects on valence and arousal

**FIGURE 2:**
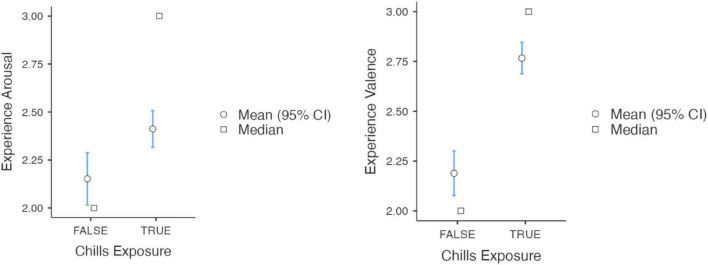
Participants rating of the experience depending on whether they experienced chills or not. We found that participants who experienced chills report significantly greater valence and arousal than those who did not.

**FIGURE 3:**
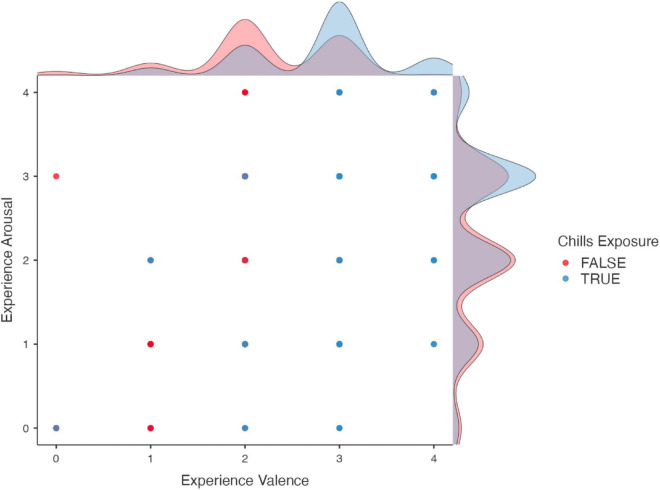
Distribution of experience rating for participants depending on whether they experienced chills or not. Participants who reported chills tend to cluster toward higher arousal and valence ratings than participants who did not.

Chills participants reported a change in valence and arousal toward the experience (Figure 2). Compared to the participants who did not report chills (*M* = 2.19, SD = 0.78), participants who reported chills (*M* = 2.77, SD = 0.78) showed a significantly more positive valence after the experience [*t*(558) = −8.3, *p* < 0.001]. We also found a change in arousal, whereby participants who reported chills (*M* = 2.41, SD = 0.94) compared to those who did not (*M* = 2.15, SD = 0.96), were more aroused [*t*(558) = −3.1, *p* = 0.002]. Hence, we reject the null hypothesis of independence between chills report and either valence or arousal (Table 3). Figure 3 shows the distribution of ratings of arousal and valence of the participants who reported experiencing chills versus who did not.

**TABLE 3:** Independent samples *T*-test for valence and arousal ratings.

			Statistic	d*f*	*P*		Effect size
Experience rating	Valence	Student’s *t*	−8.324	558	<0.001	Cohen’s d	−0.7420
Experience rating	Arousal	Student’s *t*	−3.082	558	0.002	Cohen’s d	−0.2748

**FIGURE 4:**
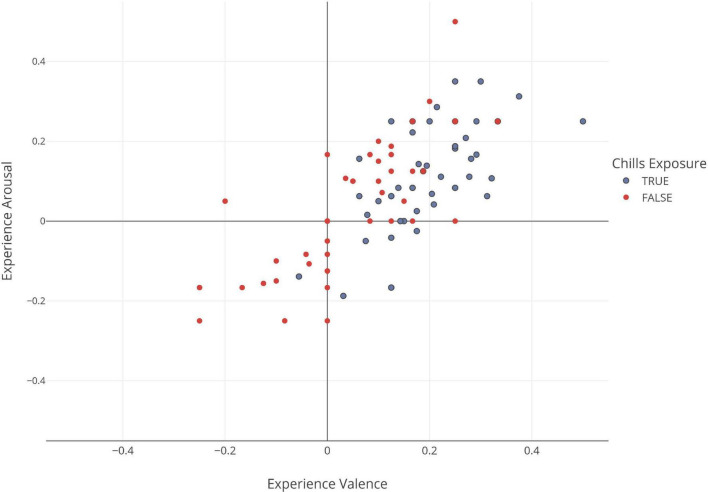
Ratings of the experience in terms of valence and arousal for each stimulus (ratings were normalized from –0.5 to 0.5). The blue dots indicate mean rating in valence and arousal for stimuli which induced chills in participants while the orange dots indicate the mean valence and arousal ratings of stimuli which did not induce chills. We see that the majority of stimuli’s ratings tend to cluster in the top right quadrant, whereas the bottom left quadrant is mostly populated by ratings of stimuli who did not cause the chills. Essentially, this suggests that the chills response influences the participant’s perception of the stimulus.

We further found the number of chills experienced by the participants to be positively correlated with the reported experience arousal (*r* = 0.104, p* = 0.014) and valence (*r* = 0.274, p^***^ < 0.001) ratings. Similarly reported chills intensity was also positively correlated with the reported experience arousal (*r* = 0.162, p^***^ < 0.001) and valence (*r* = 0.417, p^***^ < 0.001) ratings. This suggests a positive impact of the number of chills and the intensity of chills on emotional appraisal of the stimuli. Figure 4 shows the drift observed in mean arousal and valence ratings of the stimulus when participants experience chills concurrently with the stimulus versus when they do not.

**FIGURE 5:**
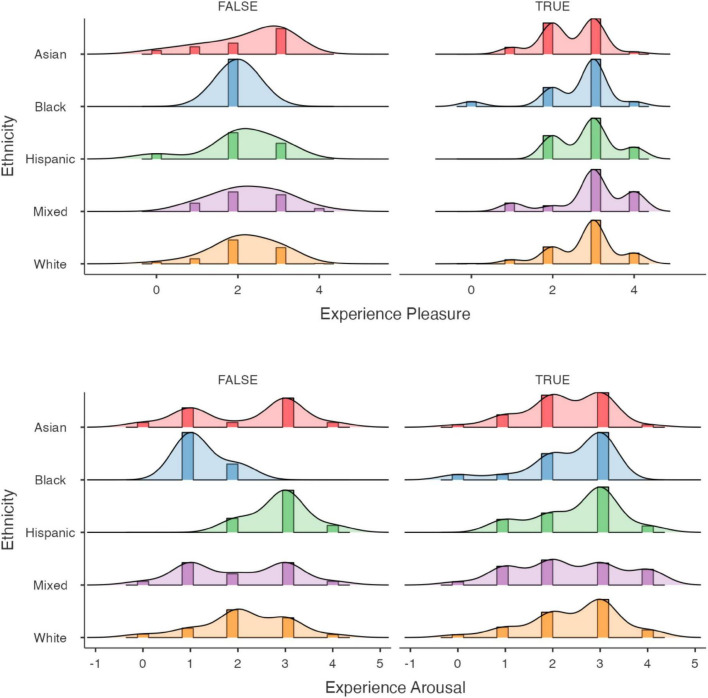
Differences in experience arousal and valence ratings across ethnicities depending on whether participants experienced chills or not. It seems that participants who reported chills show greater similarity in emotional ratings of the experience as compared to those who did not.

When examining differences across ethnicities, we found that, though initially different, the valence and arousal ratings tended toward a similar mean after the experience in chills participants (Figure 5). We note however, that this harmonization observed may be an artifact of the limited resolution of the scales and should be further investigated.

## 4. Discussion and conclusion

We examined the emotional consequences of aesthetic chills in terms of valence and arousal in a large sample of participants and across many different stimuli including music, films, and speech. We found that participants who experienced chills during the experiment reported significantly more positive emotional valence and greater arousal for their experience as compared to the participants who did not report chills. This indicates that the embodied emotion of chills causes participants to experience stimuli with greater emotional intensity. It is interesting to consider these results in light of prior research on the role of dopamine in salience signaling and so-called precision encoding (Diederen and Fletcher, 2021; Haarsma et al., 2021). Dopamine release has been linked to improved emotion recognition (Schuster et al., 2022), which may partially explain some of the results reported and call for further investigation of the chills phenomenon in dopaminergic-related pathologies (e.g., Parkinson’s disease, schizophrenia, and depression), specifically investigating the role of bodily signals in shaping the perception of the subject’s context and rewarding stimuli (Nguyen et al., 2015; Schoeller et al., 2022a).

Our findings extend previous studies by Salimpoor et al. (2011) and Laeng et al. (2016) who found that—when listening to music—physiological markers of arousal predicted chills. It is interesting to consider that Fleurian and Pearce found that chills music was on average lower in valence, and Panksepp’s found that chills were associated with perceived sadness (Panksepp, 1995), insofar as our results seem to suggest that for the same given stimulus, participants who experience chills will tend to rate it as greater in valence and arousal. The emotional drift induced by chills may explain the popularity of these stimuli in popular culture and in the web media that they were extracted from. In accordance with the existing literature, we found demographic differences in chills frequency. We found a significant difference in terms of age, as younger participants had a greater chance of experiencing chills. As a limitation, we note that this finding may be due to the age distribution in our sample, as the use of YouTube is not equally distributed in society (Auxier and Anderson, 2021) and some of the content is culture and age-specific (e.g., anime, motivation speech, and films). Our results also confirm that Item 188 of the Revised NEO Personality Inventory (NEO-PI-R) is a good predictor of chills (McCrae, 2007). However, we did not find a significant difference in terms of evaluating NEO score post exposure to chills. Furthermore, we were not able to replicate prior findings that chills vary with gender, nor did we find any ethnic difference in chills. Interestingly, when examining differences in emotion amongst various ethnicities, we found that the means for each individual ethnicity, though initially significantly different, tended to harmonize toward the same mean value. The distribution observed in Figure 5 is likely due to the five points scoring, however, we report this interesting pattern, as worthy of further research into the emotional harmonization of chills, i.e., their ability to synchronize emotional valence in a group with initial disparate ratings—putting everyone on a similar emotional level.

The capacity of chills stimuli to induce positive emotional valence and greater arousal for the same stimulus regardless of prior emotional states may be relevant for mental health intervention (Khalsa et al., 2018), e.g., to influence negatively valenced rumination in depression (Schoeller et al., 2022c), or negative affective bias in schizophrenics. The neural correlates of chills discussed in Blood and Zatorre (2001) and Salimpoor et al. (2011) suggests a pattern of activity reminding of euphoria in drug research, whereby ventral tegmental area (VTA) neurons project to the nucleus accumbens and the hippocampus in the limbic system while correlating with a deactivation of the amygdala and orbito and ventromedial prefrontal cortex (Blood and Zatorre, 2001). A future research question would be to assess whether the drift in arousal and valence also correlates with reward-driven improvement in memory encoding, as suggested by studies on the role of VTA dopaminergic projections to the hippocampus in encoding emotional memories (Shohamy and Adcock, 2010; Ripollés et al., 2018). Our results mirror those of Ferreri and colleagues on music and reward and the role of dopamine in the process (Ferreri et al., 2013, 2017, 2021, 2022). Note that activity in the insular cortex during the chills response speaks to the importance of interoception (and peripheral signals) in the chills response. Hence, while this study carried the inherent limitations of online experiments, it would be of interest to replicate the present results using peripheral measures as well as a wearable device to enhance feeling of chills by simulating the somatic markers of chills (Schoeller et al., 2019; Haar et al., 2020; Jain et al., 2022). Following the embodied model of emotions (Pezzulo, 2013), we hypothesize that such manipulation of interoceptive signals should influence further the emotional drift in valence and arousal reported by participants in this study, in line with the recent framework outlined in Schoeller et al. (2022a). Such controlled manipulations could be useful for mood disorders such as depression, specifically to address anhedonia symptoms and reward sensitivity (Webb et al., 2022).

As the first study testing ChillsDB stimuli, a number of limitations should be noted. We chose the top 50 videos of ChillsDB based on how many mentions of dictionary elements. Though specifically designed for this purpose ChillsDB is indeed not exhaustive (or even representative of the gigantic sum of data available on YouTube). Hence the results reported here, though convenient for experimental purposes, can and should be enriched by further studies. A further limitation of this study is that all measures are obtained through self-report. As emphasized previously, future studies should include physiological measures [e.g., pupil size galvanic skin response (GSR), heart rate (HR), etc.] to validate the degree to which chilling experiences are actually induced by these stimuli and confirm the present findings in terms of change in arousal.

## Data availability statement

The datasets presented in this study can be found in online repositories. The names of the repository/repositories and accession number(s) can be found below: https://doi.org/10.7910/DVN/ADLSZE.

## Ethics statement

The studies involving human participants were reviewed and approved by the Committee on the Use of Humans as Experimental Subjects at MIT. The patients/participants provided their written informed consent to participate in this study.

## Author contributions

AJ and FS conceptualized the study and designed the experiments and analyzed the results. GY and XH built the code under the supervision of AJ and FS. All authors participated equally in writing the manuscript and approved the submitted version.

## Footnotes

1
www.prolific.co
